# Recent management of endometrial cancer: a narrative review of the literature

**DOI:** 10.3389/fmed.2023.1244634

**Published:** 2024-01-03

**Authors:** George Pados, Dimitrios Zouzoulas, Dimitrios Tsolakidis

**Affiliations:** ^1^Department of Obstetrics & Gynecology, Aristotle University of Thessaloniki, “Papageorgiou” Hospital, Thessaloniki, Greece; ^2^Center for Endoscopic Surgery “Diavalkaniko” Hospital, Thessaloniki, Greece

**Keywords:** endometrial cancer, minimal invasive surgery, sentinel lymph node biopsy (SLNB), molecular classification, laparoscopy

## Abstract

Endometrial cancer is a common female gynecological neoplasia and its incidence rate has increased in the past years. Due to its predominant symptoms, most women will present uterine bleeding. It is usually diagnosed at an early stage and surgery has an important role in the treatment plan. The prognosis and quality of life of these patients can be quite favorable, if proper treatment is offered by surgeons. Traditionally, more invasive approaches and procedures were offered to these patients, but recent data suggest that more conservative and minimal invasive choices can be adopted in the treatment algorithm. Minimal invasive surgery, such as laparoscopy and robotic surgery, should be considered as an acceptable alternative, compared to laparotomy with less comorbidities and similar oncological and survival outcomes. Furthermore, sentinel lymph node biopsy has emerged in the surgical staging of endometrial cancer, in order to replace comprehensive lymphadenectomy. It is associated with less intra- and postoperative complications, while preliminary data show no difference in survival rates. However, sentinel lymph node biopsy should be offered within a strict algorithm, to avoid residual metastatic disease. The aim of this review is to analyze all the available data for the application of minimal invasive surgery in early endometrial cancer and especially the role of sentinel lymph node biopsy.

## 1 Introduction

Endometrial cancer is the third most common neoplasia in women in developed countries (1) and the sixth most common worldwide, while it holds the 14th leading cause of death in women (2). Furthermore, it is the most common cancer among gynecological malignancies (3) and in the last decades the incidence of the disease has been increasing (4, 5). Fortunately, due to its early symptoms and signs, most of the time it is confined inside the uterus and the diagnosis is made at FIGO Stage I (6), so the disease has a high 5-year survival rate (7). The prognosis is based on various factors including tumor size, histological type and grading, depth of myometrium invasion, lymphovascular space invasion (LVSI), lymph node status, disease stage, and the treatment received, including adjuvant therapies (8).

The predominant symptom of endometrial cancer is postmenopausal uterine bleeding, but abnormal uterine bleeding in pre- or perimenopausal women can also occur (9). Keeping in mind that most cases of postmenopausal bleeding are due to benign causes and only 10–15% has a malignant cause also (10). One out of eight postmenopausal women, suffering from uterine bleeding, will finally be diagnosed with endometrial cancer (11). The aforementioned data led the National Institute of Health and Clinical Excellence (NICE) to propose an endometrial biopsy for all women over the age of 45 years, during the investigation of abnormal uterine bleeding (12). All women should undergo proper clinical and gynecological examination, which are supplemented by transvaginal ultrasonography. Endometrial thickness ≥ 5 mm is the cut-off point indicating further investigation for postmenopausal women (13). The gold standard for endometrial biopsy and histological confirmation of endometrial cancer are curettage and hysteroscopy (14), but some imaging methods (CT, MRI) can provide useful information about myometrium invasion, cervical involvement, lymph node status, and regional or distant metastasis (15). New diagnostic tools (16), based on circulating cell-free DNA, have been proposed as potential biomarkers for the early detection of endometrial cancer and even atypical endometrial hyperplasia.

The definite therapeutic decision for the treatment of endometrial cancer should be made through a multidisciplinary approach and after taking into consideration the patients’ general condition, the stage of the disease and some individual risk factors. These risk factors include older age, obesity, diabetes mellitus, nulliparity, late menopause, unopposed estrogen intake, a history of breast cancer, and the use of tamoxifen (8). However, as we live in the era of personalized medicine, treatment of endometrial cancer should be tailored to each patient’s profile (17). The surgical treatment of choice for early endometrial cancer, according to the International Federation of Gynecology and Obstetrics (FIGO) (18), is total hysterectomy, bilateral salpingo-oophorectomy, and surgical staging (it may include infracolic omentectomy, lymphadenectomy, or sentinel lymph node biopsy) (19). The surgical approach for early endometrial cancer could be either a minimal invasive surgery or an open one (3, 20–22).

## 2 Surgical approach

Traditionally, laparotomy was the surgical approach of choice for endometrial cancer (23). However, minimal invasive surgery has shown great advantages for the treatment of benign gynecological diseases over laparotomy (24). Recently, minimal invasive surgery appears to be an acceptable alternative to conventional laparotomy for the treatment of endometrial cancer, because it is a safe approach with excellent surgical results, less postoperative pain and adhesions, shorter hospital stay, lower overall medical costs, and a better esthetic result (25). At present, vaginal surgery, laparotomy, laparoscopy, and robotic surgery can be used to treat endometrial cancer according to the extent of the disease (26). Historically, the first laparoscopically assisted vaginal hysterectomy was described in 1989 by Reich et al. (27) and in the same year Dargent and Salvat published the first report for laparoscopically pelvic node dissection for cervical cancer (28). Two years later, the first studies of common iliac and paraaortic lymph node dissection for endometrial cancer were published (29, 30) and more recently total laparoscopic hysterectomy without vaginal approach was described (31). In 1999, the Da Vinci robotic surgery system was developed and in 2005 Reynolds described the first case series of robotic total hysterectomies with bilateral salpingo-oophorectomy and pelvic lymphadenectomy for endometrial cancer (32).

### 2.1 Laparotomy

Generally, comprehensive surgical staging in endometrial cancer, including total hysterectomy, bilateral salpingo-oophorectomy, bilateral pelvic and paraaortic lymphadenectomy and peritoneal cytology, was accomplished via laparotomy (33, 34). This open surgical approach allowed easy identification of the common sites of metastasis (pelvic and para-aortic lymph nodes, adnexa, peritoneal surfaces, and omentum) during primary surgical treatment. However, most of the patients with endometrial cancer suffer from a metabolic syndrome, with many comorbidities like obesity, diabetes mellitus, hypertension, and heart disease. As a result, these patients suffer with significant higher peri-operative complication rates and could benefit from minimal invasive surgery (9).

### 2.2 Vaginal surgery

Vaginal procedures provide advantages concerning reduced surgical duration, and peri-operative and anesthetic morbidity, due to the absence of the incision in the abdominal wall and the possibility to avoid general anesthesia with the potential use of spinal or epidural anesthesia. However, this type of approach is not recommended for endometrial cancer, because surgical staging of the disease is not possible (9). The surgeon cannot access the nodal status or the peritoneal surfaces and must rely solely on the pre-operative imaging.

### 2.3 Laparoscopy

Laparoscopy’s role is increasing in gynecological onco-surgery for the treatment of early stages (35). A meta-analysis of four randomized controlled trials confirmed the advantages of laparoscopy compared to open laparotomy for the treatment of early endometrial cancer (21). Lower peri-operative complication rates, less blood loss, less transfusions, a shorter hospital stay and lower risk of thrombosis and/or pulmonary embolism have been described by most authors. Three large randomized trials concluded that laparoscopic hysterectomy may be as safe as abdominal hysterectomy (36), with less pain and a better quality of life for the patients (37–39), while providing economic savings (40). Many surgeons believe that lymph node dissection is not possible laparoscopically, especially in obese patients (9). However, adequate pelvic and para-aortic lymphadenectomy can be performed with the laparoscopic approach and when comparing the numbers of removed lymph nodes the results are similar (average of 18.3 nodes for laparoscopy and 17.7 for laparotomy). Especially, in obese patients most of the laparo-conversions are rarely due to technical difficulties during lymphadenectomy (41).

On the other hand, many authors have risen concerns about the possible disadvantages of laparoscopy and its impact on the survival of these patients (42). The loss of tactile sense during laparoscopy, may result in the failure to identify metastatic disease, especially high left para-aortic lymph nodes just under the left renal vein that would have been otherwise palpable during laparotomy. Moreover, there might have been a change in the patterns of recurrence sites due to the high intraabdominal pressure and the use of intrauterine manipulator, because there were insufficient data about port-site or vaginal recurrence after laparoscopic approach for the treatment of endometrial cancer (43–45). These questions about the possible effect of laparoscopy in the disease-free survival and overall survival of patients with early endometrial cancer was answered mainly by two randomized prospective trials (42, 46).

The GOG LAP2 study (42) that was published in 2012, included 2,181 patients (stage I or IIA of any histological type) that were randomly assigned to laparoscopy or laparotomy. The prior established statistical boundaries of non-inferiority, based on a 15% rate of recurrence with laparotomy, was not reached. However, the absolute percentage difference in recurrence at 3 years was 1.14% (10.24% for laparotomy and 11.39% for laparoscopy). Furthermore, the first site of recurrence was similar between the two approaches, but four trocar site recurrences were found with an incidence rate of 0.24%. The estimated 5-year overall survival was almost identical in the two treatment groups at 89.8%. In 2017, the LACE trial was published (46). This was a multinational, phase 3, randomized trial with 760 patients (stage I endometrioid type) with a primary outcome of disease-free survival and secondary outcomes of patterns of recurrence and overall survival. There was no statistically significant difference in the disease-free survival (probability at 4.5 years for laparotomy was 81.3% and for laparoscopy 81.6%) and the site of first recurrence, which was the vaginal vault (3% in each approach). Concerning recurrences in the abdominal wall, two patients had a port-site metastasis in the laparoscopy group and two patients experienced metastasis at the site of the abdominal wound in the laparotomy group. The estimated overall survival for 4.5 years was 92.4% for laparotomy and 92% for laparoscopy, with no statistically significant difference. These findings are strong evidence that laparoscopy can be an acceptable alternative to open laparotomy.

### 2.4 Robotic surgery

The advantages of robotic surgery in the treatment of endometrial cancer, compared to laparoscopy, have not been fully determined. A recent meta-analysis of 27 studies and 6,568 patients analyzed the role of robotic surgery compared to laparoscopy and laparotomy (26). When comparing robotic surgery to laparoscopy there was significantly less intraoperative complications, less blood transfusion, lower rate of conversion to open surgery, and shorter hospital stay in the robotic surgery group. Furthermore, when comparing robotic surgery to laparotomy there was significantly less blood loss and blood transfusion, less postoperative complications and shorter hospital stay. However, the operation time was significantly shorter in the laparoscopy and laparotomy group, compared to robotic surgery.

Authors attribute these advantages to the following factors: (1) 3D visualization of the operation field, allowing better detection of the vessels, thus avoiding unnecessary damage, (2) Wrist motion allows better dexterity and precision, which mimics the freedom of the human hand, and (3) Decreased surgeon’s musculoskeletal fatigue. On the other hand, the main disadvantage of robotic surgery was a longer operation time and it was mainly attributed to installation and preparation time rather than the procedure *per se* (47).

### 2.5 Natural orifice transluminal endoscopic surgery

To further minimize the morbidity of laparoscopic surgery, a recent innovation, Natural orifice transluminal endoscopic surgery (NOTES), was developed (26, 47). The basic principle of NOTES is the use of the body’s natural orifices in order to enter the abdominal cavity, eliminating skin scars and muscle or facia disruption. The first use in gynecology was reported by Ahn et al. (48) in 2012 for two ovarian cysts, but Lee et al. (49) in 2014 described the first NOTES procedure for early endometrial cancer. Although there are high-quality data of the application of NOTES for benign conditions (50), showing that it is as good as laparoscopy, its safety and feasibility has not yet been confirmed in gynecological malignant tumors. The largest study that compares NOTES with laparoscopy for early endometrial cancer is retrospective and was published in 2021 by Wang et al. (51). The study included 24 cases of NOTES hysterectomy and sentinel lymph node mapping. The results showed that NOTES is as safe and efficacious as laparoscopy for carefully selected patients with endometrial cancer. However, well designed prospective studies with longer follow-up periods are needed to verify the above mentioned results and further investigate the prognosis of these patients.

## 3 Lymph nose assessment

Surgical staging is crucial for endometrial cancer, because it defines the potential recurrence risk and the need for adjuvant therapy in high-risk patients (48, 49). When endometrial cancer has metastasized to the lymph nodes the prognosis is poor and requires the administration of adjuvant therapy (50). All patients with node positive endometrial cancer have a positive survival benefit from adjuvant chemotherapy, compared to those that will not receive, but this is not true for node negative patients (51). This fact underlines the importance of lymph node assessment during surgery, because proper surgical staging is the most important prognostic factor (52, 53).

Most patients (90%) will present early stage endometrial cancer, with no metastasis (54). However, 10–15% of them will in fact have metastatic nodal disease, while high-risk patients the percentage is up to 20% (34). Moreover, tumor grade is often upscaled from the preoperative biopsy of the hysteroscopy/curettage to the final histological specimen of the hysterectomy (55). So, it is of high importance to properly stage and treat these patients, in order to avoid missing undetected metastatic disease that may upstage them, or to avoid unnecessary full staging procedures. Unfortunately, many patients with early-stage endometrial cancer will undergo surgery with inadequate nodal evaluation (palpation of lymph nodes and biopsy only if enlarged, or completely ignored) (56). Lymph node assessment can be as low as 30% (57). The lack of surgical staging leads to unnecessary adjuvant therapy, chemotherapy, and/or pelvic irradiation, with many side effects (56). So, surgeons face the dilemma of “understating” or “overtreating” the patient numerous times.

### 3.1 Lymphadenectomy

Traditionally, there was the belief that the more lymph nodes removed, the better chance of detecting metastatic disease, but at the cost of possible side effects that the patient may develop (57). Intraoperative complications are increased during lymphadenectomy: longer operation time, excessive blood loss, vascular and nerve injury (57, 58). Lymphadenectomy is also associated with some unpleasant postoperative complications, such as lower extremity lymphedema, lymphocysts, intestinal obstruction, and deep venous thrombosis, leading to a decreased quality of life (57, 58). Some authors found that the risk of lymphedema increased to 50% when 15 or more lymph nodes were removed (59), while others stated that lymphadenectomy was an independent risk factor for lymphedema and lymphocysts (60).

Comprehensive lymphadenectomy was, and still is in some practices, an essential part of the surgical staging of the patients with endometrial cancer, because it provides information about the need of adjuvant therapy and evaluates the prognosis (61). It might also provide a therapeutic effects, because it eliminates not only existing metastases, but also occult potential metastasis (62). Large retrospective studies have shown that lymphadenectomy is associated with longer overall survival, especially in high-risk endometrial cancer (63). Moreover, concerning type II endometrial cancer, one large retrospective study (64) showed that when >20 lymph nodes where removed there was an overall survival benefit and another retrospective study (65) demonstrated that systemic pelvic and paraaortic lymphadenectomy is a significant independent therapeutic factor that prolongs disease-free and overall survival. However, two large randomized control trials showed no statistically difference in disease-free and overall survival between lymphadenectomy or not (66, 67). A more recent multicenter study (68) found that lymphadenectomy had no survival benefit in intermediate-risk endometrial cancer and another study that analyzed the SEER database demonstrated no survival difference for patients with clinical stage IA disease and any histologic grade (69).

At present, the most commonly used strategy for lymph node assessment is selective lymphadenectomy based on the “Mayo” criteria (70), meaning that lymphadenectomy can be omitted in low-risk endometrial cancer: (1) endometrioid type, (2) grade 1 or 2, (3) < 50% myometrial invasion, and (4) tumor diameter < 2 cm, and offered to high-risk endometrial cancer. Another promising predictive model for selective lymphadenectomy is the KGOG model (71). Its goal is to identify a low-risk group of patients for nodal metastasis with the evaluation of certain pre-operative criteria: (1) serum CA-125 and (2) MRI parameters (deep myometrial invasion, lymph node enlargement, and extension beyond the uterine corpus). However, nearly 80% of the high-risk group of patients with no metastases will undergo lymphadenectomy. Bases on the above mentioned facts, a less invasive procedure could offer a significant clinical value (72).

### 3.2 Sentinel lymph node biopsy

Sentinel lymph node biopsy is an image guided technique that is well established in the treatment of other cancers, such as melanoma (73) and breast cancer (74). This approach is based on the concept that lymphatic vessels drain in an orderly pattern away from the tumor to the lymphatic system. The logic of the SLNB lies in targeting the “correct”—“first” lymph nodes, that are most likely to be affected from metastatic disease, rather than removing a large number of lymph nodes for surgical staging. Therefore, if the “first” lymph node is negative for metastatic disease, then ensuing nodes should also be negative. Historically, in 1996 Burke was the first to perform SLNB in 15 cases of endometrial cancer (75).

#### 3.2.1 Technique

Many detection methods have been proposed for SLNB: blue dye method, radionuclide method, indocyanine green (ICG), carbon nanoparticle (CNP), and combination method. ICG, using near-infrared fluorescence imaging has emerged as the most recommended tracer in endometrial cancer, due to its high bilateral detection rate (76–78). The 25 mg dry powder bottle is mixed with 20 mL of sterile water in the operating room and 2–4 mL are injected at the start of anesthesia (57). However, ICG enhances the visualization of the lymphatic vessels, thus leading to an increase in “empty node” (61).

**Figure 1:**
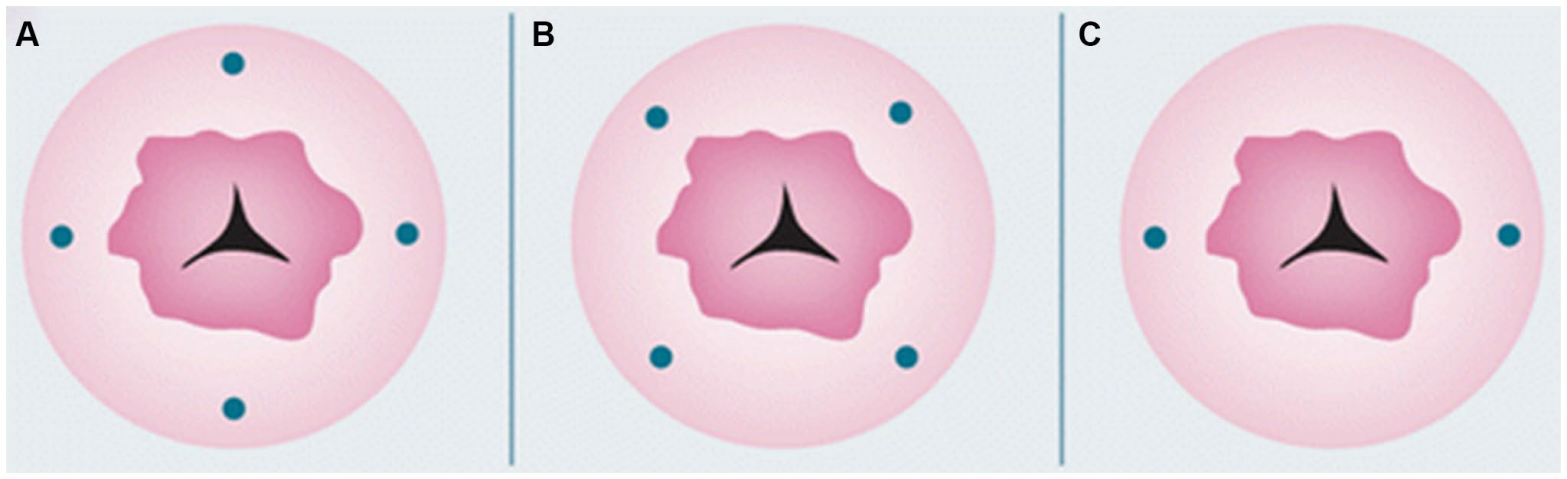
Cervical injection sites. Reprinted with permission from Zhai et al. (61), licensed under CC BY-NC.

Furthermore, there are various areas you can inject: cervical, uterine subserosa, and endometrial via hysteroscopy. Cervical injection is the most common and simplest way of injection, with a detection rate of pelvic SLNB up to 80% (79). The protocol includes a superficial (1–3 mm) and a deep (1–2 or 3–4 cm) injection at 3-, 6-, 9-, and 12-o’clock, or at 2-, 4-, 7-, 8-, and 10-o’clock points, or at 3- and 9-o’clock (Figure 1). The rationale behind cervical injection lies on the following (57): (1) the main lymphatic drainage of the uterus is from the parametria, (2) the cervix is easily accessible, and (3) the cervix in women with endometrial cancer is rarely disturbed from anatomically variations prior procedures. The main disadvantage of this method is a low para-aortic detection rate (61). This finding was also supported in a recent large meta-analysis (80), where a cervical injection showed a decreased detection rate of para-aortic SLNs compared to endometrial hysteroscopic or uterine subserosal, but without statistical significance. Furthermore, it has been shown that the true isolated para-aortic lymph node metastases is even lower after ultra-staging of the “false negative” pelvic lymph nodes and the discovery of micrometastases or ITCs (81). On the other hand, the two main lymphatic drainage pathways [upper paracervical pathway (UPP) with draining medial external and/or obturator lymph nodes and a lower paracervical pathway (LPP) with draining internal iliac and/or presacral lymph nodes] were identified irrespectively of the injection site in a recent well-designed prospective trial (82).

#### 3.2.2 Diagnostic accuracy

Sentinel lymph node biopsy is supposed to have high sensitivity and low false negative rate. A detection rate of 80–90% or greater is preferred (83). Centers are required to perform in the beginning SLNB followed by lymphadenectomy, in order to assess their indicators. Implementing an SLNB algorithm significantly reduces the false negative rate of the procedure. The Memorial Sloan Kettering Cancer Center (MSKCC) SLNB algorithm improves sensitivity from 85.1 to 98.1% and negative predictive value from 98.1 to 99.8% (57). The algorithm includes (84): (1) peritoneal and serosal evaluation and washing, (2) retroperitoneal evaluation, all suspicious enlarged lymph nodes should be removed, (3) if SLN mapping fails, a side-specific lymphadenectomy (pelvic, common iliac, and interiliac) should be performed at the side of the mapping failure, (4) Surgeons can decide on para-aortic lymphadenectomy, and (5) ultra-staging pathology should be performed after the operation. However, a new predictive score has been proposed to omit lymphadenectomy to certain patients if SLNB fails (85). This model includes myometrial infiltration, tumor grading, tumor diameter, and CA125 assessment.

There are some factors that may affect the diagnostic value of the SLNB. Firstly, the surgeons experience plays a center role in the detection rate. An experience of 30 or more SLNB procedures is required as a learning curve, with an increase from 78 to 94% (83). Second, tracer type and injection site are equally important. The disadvantage of ICG, empty node, can be decreased by a combination method (86) and cervical reinjection can be used when mapping is failure occurs (81, 87). Detection rate can, also, be affected from the patient’s age, BMI > 40 (obesity), pelvic anatomically abnormality, pelvic adhesions (operation or history of radiation), and lymphatic vessels obstruction (tumor metastasis or deep myometrial infiltration) (78, 88). Ultra-staging pathology is crucial for the detection of low volume lymph node metastases. Other factors, such as lymph vascular space invasion (LVSI) or non-endometrioid histology are considered as independent risk factors, but are lacking strong evidence (89).

#### 3.2.3 Ultra-staging pathology

**Figure 2:**
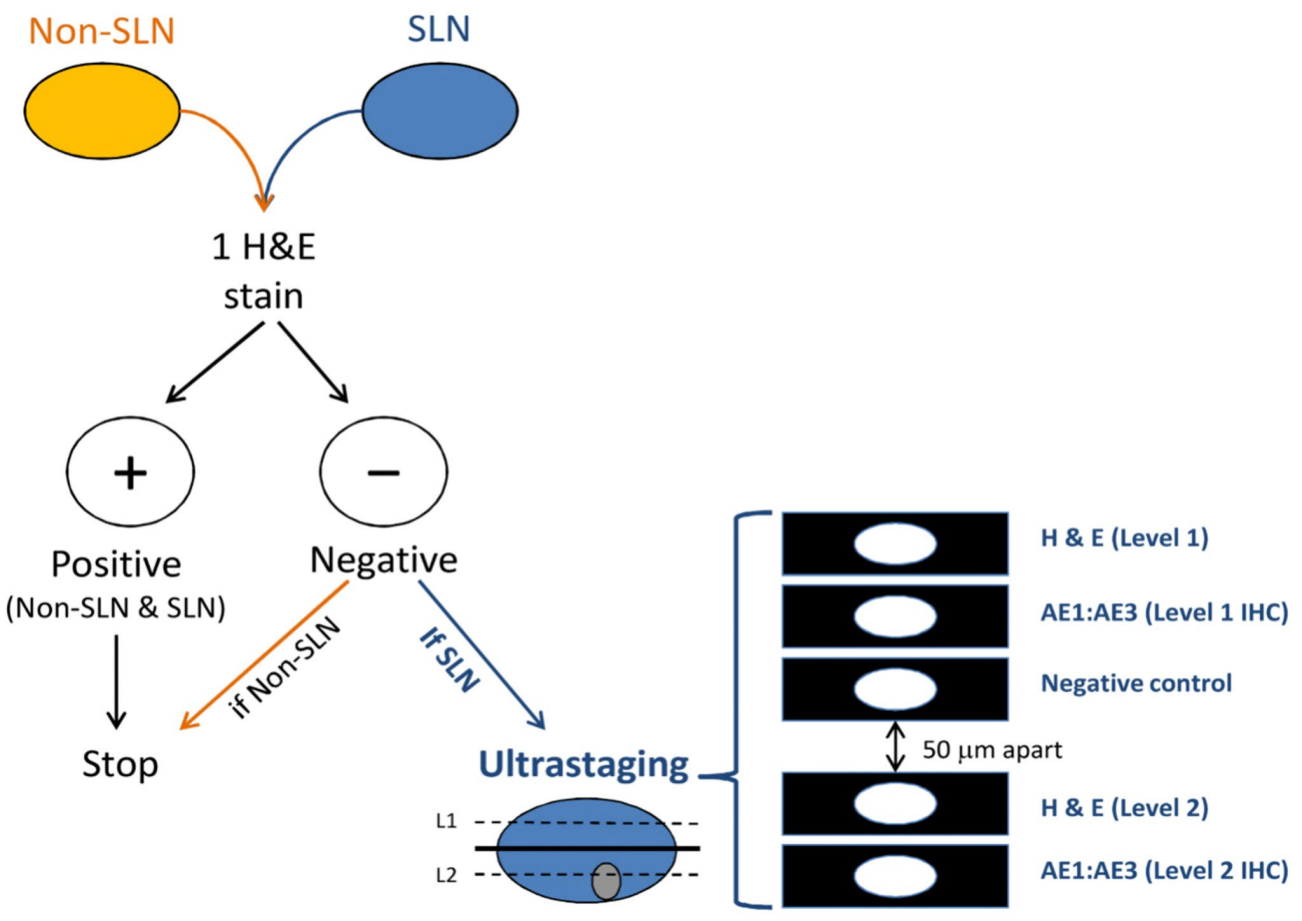
MSKCC ultra-staging protocol. Reprinted with permission from Wolters Kluwer Health, Inc., Copyright © 2017 by the International Society of Gynecological Pathologists (93).

**Figure 3:**
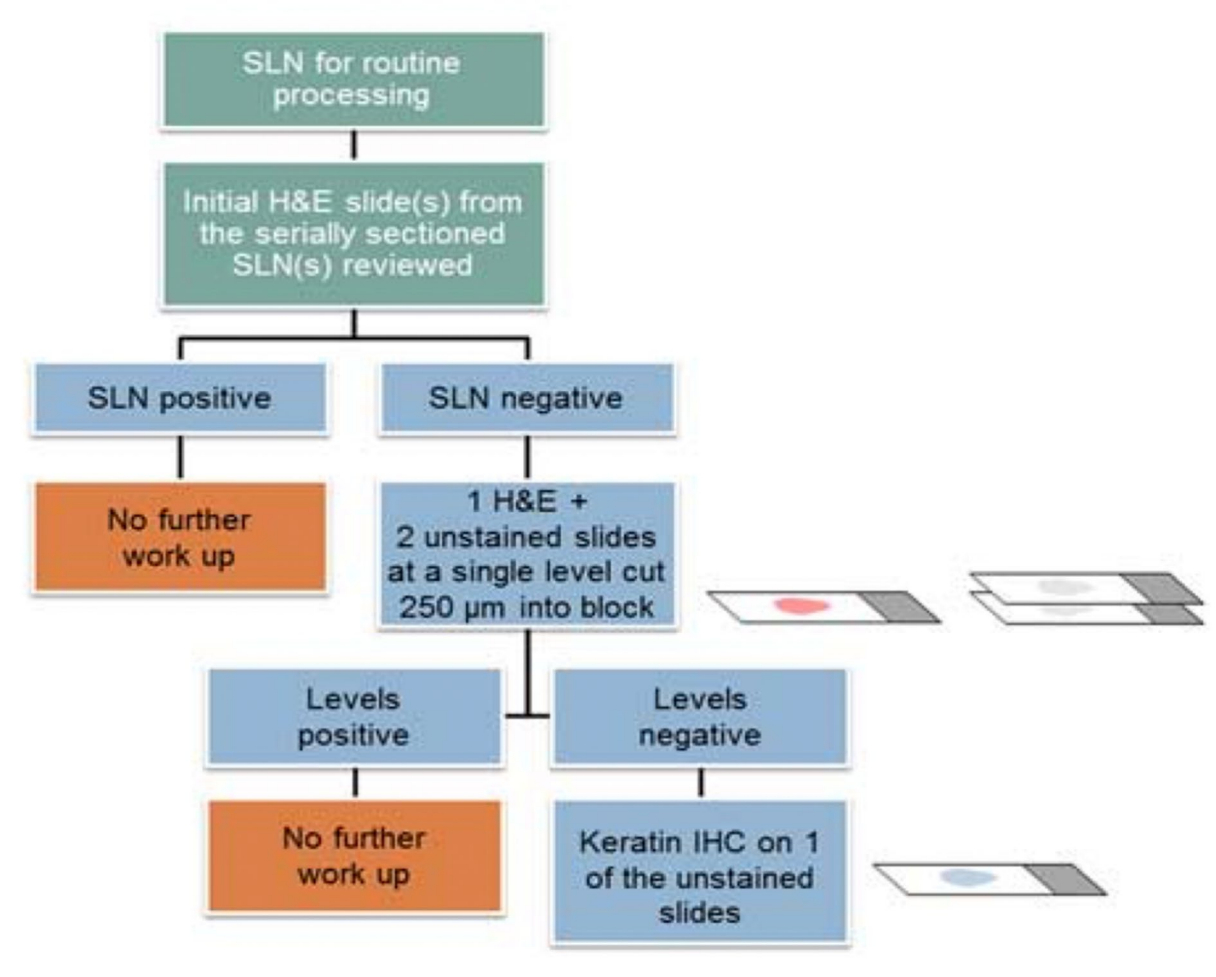
M.D. Andersson Cancer ultra-staging protocol. Adapted from Wolters Kluwer Health, Inc., Copyright © 2018 International Society of Gynecological Pathologists. Published by Wolters Kluwer Health, Inc. on behalf of the International Society of Gynecological Pathologists (94).

The goal of ultra-staging is to identify low volume metastatic disease (LVMD) in the SLNB (90) and for this purpose H&E and IHC staining is used. The standards for LVMD were based on breast cancer guidelines (91): (1) macro-metastases (> 2 mm), (2) micro-metastases (0.2–2 mm), and (3) isolated tumor cells (ITCs; < 0.2 mm). ITCs are considered as node negative disease, pN0(i+). There is no standardized protocol for ultra-staging yet. MSKCC protocol (92) (Figure 2) divides H&E negative SLN into two levels (50-μm apart). If still the H&E staining remains negative, two consecutive 5-μm thick sections are sliced at every level, one for H&E and one for IHC. Differently, M.D. Andersson Cancer Center protocol (93) (Figure 3) cuts three serial 250-μm thick sections in an H&E negative SLN. Slicing H&E staining is repeated and if still negative, the other two slices undergo IHC staining. There is no difference between the two protocols concerning the detection of infiltrated SLN in low- and high-risk endometrial cancer patients (94). Interestingly, LVMD (micro-metastasis and ITCs) account approximately for 50% of all positive SLNB that underwent ultra-staging (61). The use of IHC improves the detection of SLNB metastasis by two times compared to H&E. Possible disadvantages of ultra-staging is the fact that is time consuming and cannot be done during the operation.

#### 3.2.4 Therapeutic safety

The main prospective trials that established the therapeutic value of SLNB are three. In 2017, the FIRES trial (95) was first published. It was a multicenter, prospective, cohort study contacted in America and included 340 patients with any histology clinical stage I disease. All patients underwent pelvic lymphadenectomy after SLNB, but para-aortic lymphadenectomy was something that surgeons decided on. The results of the study showed patients that a SLNB negative for metastatic disease is accurate in more than 99% of the cases (sensitivity) and only 3% of the cases with nodal involvement will not be recognized from SLNB (false-negative rate). Moreover, the SHREC trial (81) was published in 2019. It was a prospective, non-randomized trial from Sweden that included 257 patients, who underwent both pelvic and para-aortic lymphadenectomy after SLNB. The sensitivity of the study was 98% and the negative predictive value was 99.5%. Finally, in 2021, the SENTOR study (96) was published. It was a prospective, multicenter, cohort study from Canada, which include 156 patients with intermediate- and high-risk endometrial cancer. Para-aortic lymphadenectomy was performed only in high-risk patients. More than 96% of the patients with node positive disease was identified from SLNB and 99% with node negative SLNB had truly node negative disease.

The impact of SLNB on long-term prognosis of endometrial cancer patients has been of great concern among authors. Still, future randomized control trials with long-term follow-up are needed to fully reveal the oncological outcomes of SLNB. However, several studies have shown promising results about prognosis (97). A recent meta-analysis comparing lymphadenectomy and SLNB showed no difference in recurrence rate of para-aortic lymph node metastasis (98). The same results were found in a large cohort (99), where lymphadenectomy failed to improve disease-free and overall survival. The most recent multi-institutional retrospective study (100), comparing SLNB, SLNB + lymphadenectomy and lymphadenectomy alone found no difference concerning disease-free survival and demonstrated similar survival rates among all risk groups.

An important advantage of SLNB is reduced intraoperative and postoperative complications of lymphadenectomy. Accorsi et al. (101) found that SLNB reduces the risk of intraoperative and postoperative complications, while Pearson et al. (81, 102) demonstrated that SLNB reduces lower extremity lymphedema by 14 times. Similar results are presented from several recent meta-analyses and indicate that SLNB can improve the quality of life of these patients (103, 104).

Last but not least, there are some concerns regarding SLNB in endometrial cancer. The first concern is about the need of para-aortic lymph node dissection, which is currently decided on by the surgeons. It is proven that endometrial cancer can directly metastasize to the para-aortic lymph nodes through the pelvic-infundibular ligament pathway. The incidence of para-aortic metastases is 51% when pelvic lymph nodes are positive, but it dramatically decreases to 3% when pelvic lymph nodes are negative (61). Some authors have proposed the implementation of PET-CT in the preoperative set-up for the assessment of the para-aortic space (105, 106), however the survival benefit from para-aortic lymphadenectomy remains controversial. The above-mentioned problem for the SLN in the para-aortic space could be addressed by using other tracer injections sites or a combination of them. Some authors have proposed the transvaginal ultrasound-guided myometrial injection of radiotracer (TUMIR) (107), which have shown detection rates higher that 45%, while other studies (108, 109) described the use of dual tracer injection at both the cervix and the uterus fundus, with adequate mapping in the pelvis and the aortic space. In 2020, Martinelli et al. (110) presented a large retrospective study showing that hysteroscopic tracer injections leads to a higher SLN detection rate in the para-aortic area. The second major challenge when preforming SLNB is the risk of possible residual metastasis to non-SLN. The risk is associated with the size of the SLN metastasis and uterine higher-risk factors (111). Therefore, it is of high importance to carefully follow the SLNB algorithm and always remove any suspicious enlarged lymph nodes.

## 4 Molecular classification

Moreover, in the era of the new molecular classification of endometrial cancer questions have arisen about its implementation not only in the planning of the adjuvant treatment (e.g., MMR deficient endometrial carcinomas respond to immune therapy), but also for the surgery planning and especially the lymph node staging (58). The diagnostic algorithm of the molecular features of the tumors includes the immunohistochemical marker p53, the molecular analysis of the exonuclease domain of POLE and the mismatch repair (MMR) status [either with the four major immunohistochemical markers MLH1, PMS2, MSH2, and MSH6 or the molecular analysis for microsatellite (MSI) status] (59). Alternatively, a two-marker approach can be used (PMS2 and MSH6) to detect MMRd, because there will always be loss of PMS2 expression in absence of MLH1 and MSH6 will always be lost in absence of MSH2. But, in cases of PMS2 or MSH6 loss, MLH1 and MSH2 should be also performed (60). In 2016, Talhouk et al. (61) showed that molecular classification from the pre-surgery endometrial samples can accurately predict the molecular features of the final hysterectomy tumor, with even higher concordance than grade and histotype. This information during the initial diagnosis could possibly alter the surgical management plan and also help to carefully choose patients that will undergo fertility-sparing (62). Patients with favorable molecular features could be spared from any lymph node staging technique and high-risk patients could be offered more radical surgical lymph node staging.

On the other hand, adjuvant therapy offered to endometrial cancer patient’s needs re-evaluation, because the new molecular classification could possibly change the offered treatment (monitoring or radiotherapy or chemotherapy or immunotherapy). However, further trials evaluating treatment effectiveness within biologically similar tumors and enhance outcomes in this disease site should be of high priority to improve adjuvant therapy in endometrial cancer (112). A treatment algorithm based on histopathological features compared to an algorithm based on molecular features have completely different treatment strategies (113). An ongoing randomized trail (PORTEC-4a) (114) and an ongoing prospective trial (RAINBO) (115) will provide useful information about de-escalation of adjuvant treatment and its impact on survival and quality of life of endometrial cancer patients.

## 5 Conclusion

Summarizing, the use of minimal invasive surgery is the approach of choice for the management of early endometrial cancer. No clear consensus has been made concerning the choice between laparoscopy or robotic surgery, so both approaches are proposed, based on the experience of the surgeon and the availability of a robotic platform. However, laparotomy remains a viable choice for some special cases with the same oncological results, but with possible higher post-operative complications. On the other hand, SLNB is the gold-standard for the lymph node staging for early endometrial cancer, but its use for high-risk patients is still controversial. So, lymphadenectomy still plays an important role, but systematic lymphadenectomy for diagnostic purposes should no longer be performed. Careful patient selection should be made, in order to avoid over-treatment, but especially under-treatment. Lastly, ultra-staging of the SLNs is extremely important to identify all possible lymph node metastases and better plan the adjuvant treatment of these patients. Table 1 summarizes the surgical approaches and the lymph node staging techniques.

**Table 1:** Management of early endometrial cancer.

Surgical approach	
Laparotomy	Acceptable alternative of MIS Better visualization of the whole abdomen High peri-operative complications Excellent oncological outcomes
Vaginal	Not recommended for EC
Laparoscopy	Gold-standard Low peri-operative complications Loss of palpation feeling of enlarged lymph nodes Same oncological outcomes as laparotomy
Robotic	Gold-standard (superiority over laparoscopy not clear) Low peri-operative complications Loss of palpation feeling of enlarged lymph nodes Same oncological outcomes as laparotomy
NOTES	Promising technique Low peri-operative complications Loss of palpation feeling of enlarged lymph nodes Further high-quality data needed for the oncological outcomes
**Lymph node staging**	
Lymphadenectomy	Acceptable alternative of SLNB Recommended for high-risk patients High peri-operative complications Excellent oncological outcomes
SLNB	Gold-standard Low peri-operative complications Controversary for high-risk patients Excellent oncological outcomes, but with ultra-staging

The European consensus of ESGO/ESTRO/ESP published in 2021, the updated guidelines (116) on the management of endometrial cancer and proposed the use of minimal invasive approach and SLNB:

- Minimal invasive surgery is the preferred surgical approach, including high-risk patients.
- Any intraperitoneal tumor spillage, including tumor rapture or morcellation (even in a bag) should be avoided.
- If vaginal extraction risk uterine rapture, other measures should be considered (mini-laparotomy and use of endobag).
- Relative contraindications for minimal invasive surgery are extrauterine tumor spread (excluding lymph node metastases).
- Sentinel lymph node biopsy can be considered for low- and intermediate-risk patients. It can be omitted in the absence of myometrial invasion. Lymphadenectomy is not recommended.
- Sentinel lymph node biopsy is an acceptable alternative to lymphadenectomy for high-intermediate- and high-risk patients, where surgical staging should always be performed.
- If SLNB is performed:
  1. ICG with cervical injection is the preferred technique.
  2. In case of no visualization tracer, re-injection is an option.
  3. Side-specific lymphadenectomy should be performed in high-intermediate- and high-risk patients, when SLN is not detected in either pelvic side.
  4. Ultra-staging pathology is recommended.

## Author contributions

GP: conceptualization and writing—review and editing. DZ: writing—original draft preparation, methodology, and software. DT: supervision and writing—review and editing. All authors contributed to the article and approved the submitted version.
